# Quality of Root Canal Filling in Curved Canals Utilizing Warm Vertical Compaction and Two Different Single Cone Techniques: A Three-Dimensional Micro-Computed Tomography Study

**DOI:** 10.30476/dentjods.2023.98119.2054

**Published:** 2024-06-01

**Authors:** Yazdan Shantiaee, Babak Zandi, Mohammadreza Hosseini, Paria Davoudi, Mehran Farajollahi

**Affiliations:** 1 Dept. of Endodontics, School of Dentistry, Shahid Beheshti Medical University, Tehran, Iran; 2 Endodontist, Dept. of Endodontics, School of Dentistry, Shahid Beheshti Medical University, Tehran, Iran; 3 Endodontist, Private Practice, Tehran, Iran; 4 Postgraduate Student, Dept. of Endodontics, School of Dentistry, Shahid Beheshti Medical University, Tehran, Iran

**Keywords:** AH Plus sealer, Micro-CT, Single cone technique, SureSeal sealer, Warm vertical compaction technique

## Abstract

**Statement of the Problem:**

Successful endodontic treatment depends on three-dimensional (3D) root canal filling to prevent the leakage of residual bacteria. Different obturation techniques with different sealers should be compared by employing advanced assessment tools.

**Purpose:**

This study compared the obturation quality of warm vertical compaction (WVC) and two different single-cone (SC) techniques using micro-computed tomography (micro-CT).

**Materials and Method:**

Thirty-three extracted maxillary molars with mesial root canal curvature of 20‒40º were prepared *in vitro* with One-Curve files and randomly assigned to three groups (n=11) for root canal obturation with WVC, SC technique with AH Plus sealer (SC-AH), or SC technique with SureSeal bioceramic (BC) sealer (SC-Su). The root canals underwent micro-CT examinations before and after obturation to determine the volume percentages (VPs) of voids and gaps at different distances from the apex (coronal, middle, and apical thirds). Data were analyzed by Kruskal-Wallis and Mann-Whitney U tests.

**Results:**

The highest VP of voids and gaps was recorded in the SC-Su group, with no significant difference from other groups (*p*> 0.05). There were significant differences in VP of gaps at different distances from the apex (*p*<0.05). The VPs of gaps in the apical third of all the samples were significantly higher than in the coronal and middle thirds. However, the difference in VP of voids was not significant at different distances from the apex (*p*>0.05).

**Conclusion:**

None of the tested techniques could provide a void-free and gap-free filling. The apical third of the canals showed the highest VP of gaps in all obturation techniques.

## Introduction

One principal aim of endodontic treatment is to obturate the root canal system three-dimensionally and provide a tight seal to prevent the leakage of residual bacteria and their toxins and reinfection [ 1 - 2 ]. The root canal obturation quality affects the treatment success, and the absence of voids in the root canal obturation, especially in the middle and apical thirds, is associated with improved outcomes [ 3 - 4 ]. For this purpose, different root canal-filling materials and techniques have been developed to achieve ideal obturation and completely adapt obturating materials with the dentinal walls of the root canal system [ 5 ].

The benefits of thermoplastic obturation techniques, including warm vertical compaction (WVC), have been well documented, especially for treating irregular or oval-shaped root canals. Such techniques were introduced to improve obturation homogeneity [ 6 ]. The single-cone (SC) technique has become popular, using the best-matched gutta-percha point after root canal preparation with rotary systems. It is faster than the lateral compaction technique, decreases the pressure applied to root canal walls [ 7 - 8 ], and yields the same obturation quality as the thermoplasticized techniques [ 2 ]. However, some studies have questioned the sealing properties of this method, especially in irregular canals, due to the excess sealer surrounding the master cone [ 7 , 9 - 10 ]. This drawback was overcome by the advent of bioceramic (BC) sealers due to their optimal dimensional stability and superior adaptation [ 11 - 12 ]. BC sealers are biocompatible, antibacterial, and stable within biological environments and improve the outcomes of endodontic treatments [ 13 ]. 

Obturation quality and homogeneity are evaluated by the number of voids in the root canal filling material [ 14 ]. Among the available techniques to assess the obturation quality, X-ray micro-CT (micro-computed tomography) is more popular than others since it is a non-invasive and non-destructive technique, allowing three-dimensional (3D) evaluation of the root canal filling [ 15 ].

Despite the advantages of micro-CT, relatively few studies have been conducted so far to compare the obturation quality of WVC and SC obturation techniques using BC sealers, especially in curved canals. Therefore, the present study compared the obturation quality of WVC and SC techniques with two different sealers using micro-CT.

## Materials and Method

### Ethics approval

The Ethics Committee of Shahid Beheshti University of Medical Sciences approved the study protocol (IR. SBMU.DRC.REC.1397.040).

### Sample collection

Thirty-three mature, sound, extracted molars with mature roots and mesial root curvature of 20‒40º, as measured by the Schneider method [ 16 ] according to the radiographic buccolingual and mesiodistal dimensions, were included in this study. Care was taken to select teeth with the same root length to standardize the study sample regarding the root length. The teeth were inspected under 12× magnification (Stemi SV6; Carl Zeiss, Oberkochen, Germany), and the roots with resorption defects or caries below the cementoenamel junction, fractures, or cracks were excluded. The tooth samples were stored in 5.25% NaOCl solution for 24 hours to eliminate the periodontal ligament tissues, rinsed with water, and stored in 0.2% sodium chloride and thymol solutions.

### Root canal preparation

An experienced operator carried out all clinical procedures. After preparing the access cavity by a diamond fissure bur and negotiation of root canal orifices, first, the patency of apical foramen was ensured by introducing a #10 stainless steel K-file (Dentsply Tulsa Dental, Tulsa, OK, USA) into the root canal. After the file tip became visible at the apex under a stereomicroscope, 1 mm shorter than this length was recorded as the working length (WL). Before shaping the canals, the coronal structure was removed at a 4-mm distance from the bottom of the pulp chamber by an Endoflare file (Micromega, France) at a speed of 400 rpm and 3N/cm torque. A One-G file (Micromega. France) was then used at 400 rpm and 1.2N/cm torque to create a glide path. Then, the canals were shaped using the One-Curve file (Micromega, France) at 300rpm and 2.5N/cm torque. After two pecking movements, the flute of the files was cleaned with moist gauze. Then the root canals underwent an irrigation procedure with 5mL of 1% NaOCl using a 30-gauge double-vented, close-ended irrigation needle. The smear layer was removed by irrigating the root canals with 5mL of 17% EDTA for 2 minutes, 5mL of 5.25% NaOCl, and a final saline rinse.

### Micro-CT imaging

**Figure 1:**
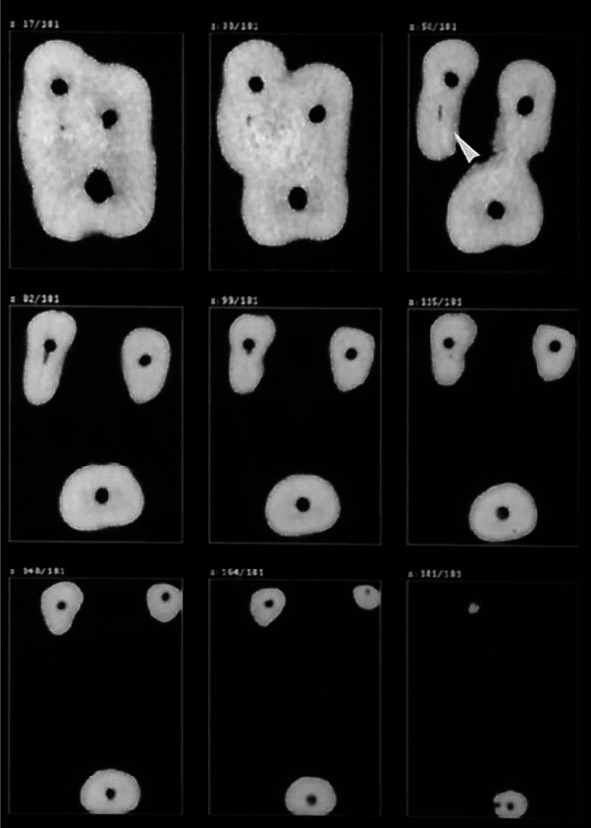
Micro-CT images of the canals after preparation (axial view)

All the roots were scanned after instrumentation and after obturation by a micro-CT scanner (LOTUS- in vivo, Behin Negareh Imaging Technologies Development Company, Iran). For this purpose, the teeth were mounted in acrylic resin with the access cavities facing down to facilitate precise repositioning and reproducible imaging. Seven-tooth groups were positioned in a single sample holder and transferred to the carbon-fiber bed. The teeth were scanned with the exposure settings of 90 kV, 200 mA, 360° rotation, 500 ms exposure time, and an 0.5 rotation step, with a 30-µm voxel size (Figure 1). A copper filter with 0.1-mm thickness was used. The 3D reconstruction of images was conducted with Amira software version 6.0 (FEI Experimental Science Group, SAS, Burlington, MA) MeVisLab software version 6.4 (MeVis Medical Solutions AG) was applied for 3D volumetric visualization and root canal measurements. The volume of the prepared root canal area in each slice was determined from the root canal orifice to the apical constriction. The root canal volume in each segment was achieved by multiplying the root canal area by the slice thickness (0.5mm). The root was sectioned into three equal coronal, middle, and apical thirds. Then each segment’s volume was calculated separately.

### Root canal obturation

After obtaining 3D images of the prepared canals, the teeth were randomly divided into three groups (n=11) as follows.

### Group 1: WVC

The root canal walls were coated with AH Plus sealer (Dentsply International Inc, York, PA) using a Lentulo spiral. Then a matched gutta-percha point was sealer-coated and inserted into the canal within 1mm of the WL. A #40 heated plugger (SP1, Fanta-dental, China) was used to pack down gutta-percha to 4mm of the WL, and the canal was then backfilled with Meta BioMed (Korea) obturator with 3-4-mm gutta-percha segments until complete obturation of the canals was achieved.

### Group 2: SC technique with AH Plus sealer (SC-AH)

AH Plus sealer was applied to the root canal by a Lentulo-spiral. A proper-size gutta-percha point was sealer-coted, inserted into the canal, and gently moved with up and down motions three times to enhance sealer distribution in the canal. Excess filling material was then cut 1 mm above the orifice using a heated plugger and packed vertically using a cold plugger.

### Group 3: SC technique with SureSeal sealer (SC-Su)

SureSeal sealer (Sure Dent Corporation, Gyeonggido, South Korea) was injected into the canal by a 24-gauge needle tip provided by the manufacturer. A proper-size gutta-percha point was sealer-coated and inserted into the canal gently with up and down motions three times to enhance sealer distribution in the canal. Excess filling material was then cut 1mm above the orifice using a heated plugger and packed vertically using a cold plugger. The pulp chamber was cleaned with a cotton pellet soaked in 70% ethanol in all the groups, and the access cavity was sealed with Coltosol (Ariadent, Iran). The teeth were incubated at 37°C under 100% humidity for 72 hours to allow the complete setting of sealers in the canals before post-obturation scanning. The teeth then underwent micro-CT again. 

### Image analysis

In this study, voids were defined as bubbles or empty spaces within the root filling, while gaps were defined as the spaces remaining at the interface of root-filling material and root canal walls.

**Figure 2:**
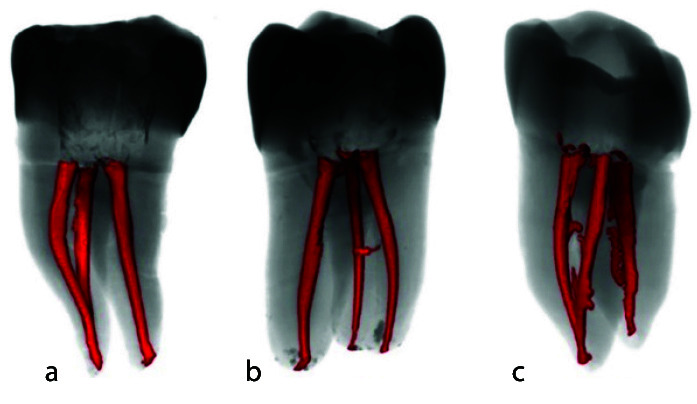
Representative three-dimensional reconstructions of specimens after obturation. **a:** Single-cone SureSeal bioceramic sealer (SC-Su) group, **b:** Single-cone AH plus sealer (SC-AH) group, **c:** Warm vertical compaction (WVC) group

**Figure 3:**
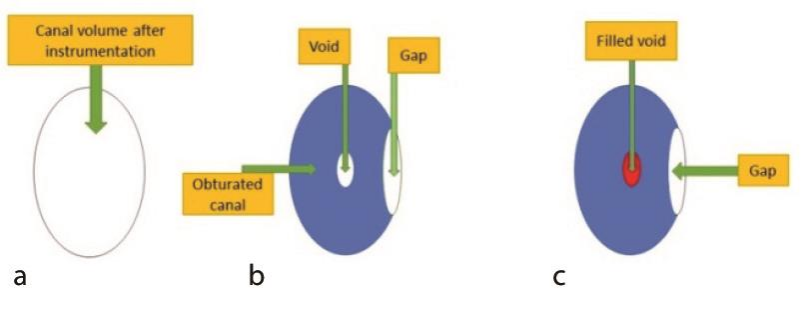
**a:** Root canal volume after instrumentation. **b:** Void, gap, and obturated canal. **c:** The volume of voids was calculated by subtracting the volume of the obturated canal with filled voids from the obturated canal. The volume of gaps was calculated by subtracting the canal volume after instrumentation from the volume of the obturated canal with filled voids

**Figure 4:**
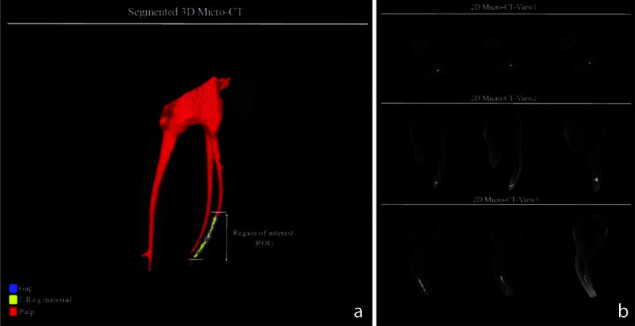
**a:** The segmented 3D micro-CT image for one of the samples from group 2; voids/gaps (blue). The filling material (yellow) and pulp (red), **b:** The 2D micro-CT images for one of the samples from group 2 in different views

The following parameters (mm^3^) were measured on micro-CT scans by the Image J version 1.49 software (National Institutes of Public Health): (1) the root canal volume, (2) the volume of voids distributed within the filling material, and (3) the volume of gaps along the canal walls at the root filling-canal wall interface (Figure 2). For this purpose, the entire root canal volume and root filling volume were first measured. Next, the voids in the root filling were filled by the software to calculate the whole root-filling material’s volume with filled voids. This volume was subtracted from the volume of the entire canal to calculate the volume of gaps. Next, the volume of voids within the root-filling material was calculated by subtracting the root-filling material’s volume from the volume of the root-filling material with filled voids (Figure 3). The volume of voids and gaps was expressed as a percentage of root canal space after measurement (Figure 4).

### Statistical analysis

SPSS 24 was used for statistical analyses. Since the data were not normally distributed, statistical analysis was conducted by the nonparametric Kruskal-Wallis and Mann-Whitney U tests. The significance level was defined at *p*<0.05. 

## Results

Tables 1 and 2 present the mean volume percentages (VP) of voids and gaps in the study groups in the coronal, middle, and apical thirds. The highest VPs of voids were observed in the apical third and total root canal space in the SC-Su group, with no significant difference from other groups (*p*> 0.05). Also, the highest VP of gaps was recorded in the group 3(SC-Su group), with no significant difference from other groups (*p*> 0.05) of gaps in the apical thirds of all the canals (n=33) were significantly higher than those in the coronal and middle thirds in all the obturation techniques (Table 3). However, the differences in the VPs of voids were not significant at different distances from the apex in any group (*p*> 0.05). 

**Table 1:** Means and standard deviations of the volume percentages (VPs) of voids in each group at different levels from the apex

Groups	Total	Coronal third	Middle third	Apical third
WVC	0.70	0.82 (0.59)	0.56 (0.21)	0.74 (0.31)
SC-AH	0.63	0.68 (0.27)	0.52 (0.23)	0.72 (0.31)
SC-Su	0.93	0.54 (0.28)	0.96 (0.53)	1.29 (0.59)

**Table 2:** Means and standard deviations of the volume percentages (VPs) of gaps in each group at different levels from the apex

Groups	Total	Coronal third	Middle third	Apical third
WVC	4.30	4.16 (0.21)	3.08 (0.52)	5.66 (0.58)
SC-AH	4.51	3.84 (0.36)	4.62 (0.29)	5.14 (0.33)
SC-Su	4.94	4.29 (0.31)	4.57 (0.23)	5.85 (0.31)

**Table 3:** The volume percentages (VPs) of voids and gaps at different levels from the apex

Level	VP of voids	VP of gaps
Coronal third	0.68	4.10
Middle third	0.68	4.10
Apical third	0.92	5.57*

* Statistically significant

There were significant differences in the VPs of gaps at different levels from the apex (*p*< 0.05), and the VPs

## Discussion

Optimal void-free obturation of the root canal system is associated with a higher success rate of endodontic treatment. However, achieving a void-free obturation is almost impossible [ 17 - 19 ]. The presence of gaps at the root canal-filling material‒canal wall interface is more likely to affect the outcome than voids within the root-filling material, as the former can lead to leakage and direct contact of bacteria with the canal walls. Most previous studies on the quality of obturation of different techniques have examined straight canals, and less attention has been paid to curved canals and BC sealers [ 17 - 19 ]. Therefore, the present study evaluated the curved mesial canals of extracted molars to better simulate the clinical setting. The presence of gaps and voids is influenced by various factors such as the clinician’s experience, sealer characteristics, root canal anatomy, and type of obturation technique [ 20 ]. In the present study, none of the tested techniques yielded a void- and gap- free obturation, consistent with previous studies [ 21 - 22 ].

The WVC technique has been reported to provide a more homogeneous filling compared to the SC and cold lateral compaction techniques. In fact, the simplicity of the SC technique has been reported as its main advantage [ 23 ]. However, some studies have not reported any additional advantages for obturation with the SC technique [ 24 ]. Alshehri *et al*. [ 25 ] demonstrated no significant difference in obturation quality between the WVC and SC techniques in the apical third of curved mesial canals, consistent with the present findings. Another study by Keles *et al*. [ 2 ] reported similar, but not void-free, obturation quality of the two techniques in band-shaped isthmi. As stated by Hörsted-Bindslev *et al*. [ 26 ], the root canal anatomy has the greatest impact on the filling quality, which probably explains the discrepancy in the results, as the anatomy of root canals is highly variable and complex. In addition, different sealers and obturation materials affect the number of inevitable voids and gaps [ 27 ]; according to Penha da Silva *et al*. [ 28 ], there were no significant differences in void volumes between the SC and cold lateral compaction techniques using EndoSequence BC sealer in oval straight canals.

The present study showed no significant difference in the VP of voids and gaps between the experimental groups. Consistent with the present findings, Celikten *et al*. [ 6 ] reported no significant difference in the percentage of voids between the EndoSequence BC sealer and Smartpaste Bio. In the present study, the apical, middle, and coronal thirds of the canals were separately assessed since voids and gaps in each third are clinically significant and can predict the behavior of different filling methods and sealers in different anatomical areas.

The lack of a significant difference between the obturation groups may also be explained by the heat source that only reaches 5 mm of the working length during the packing process in WVC [ 1 ]. Although it is recommended to insert the heat carrier by 3-5 mm of the WL in the WVC method [ 29 ], it has been reported that only 1‒2 mm of the apical gutta-percha undergoes plastic deformation due to the heat generated by the heat carrier [ 30 ]. Hence, the SC technique fills the 2‒4 mm of the apical portion of the canals in all obturation groups [ 24 ]. Li *et al*. [ 24 ] compared three obturation techniques of GuttaCore, WVC, and cold lateral compaction by micro-CT. They assessed the VPs of gaps and voids at three canal levels (0-4mm, 4-8mm, and 8-12mm from the WL). They reported the lowest VPs of gaps and voids in canals filled with GuttaCore and WVC. However, the root lengths were not standardized in their study, which could be concerned as a limitation of their study. Accordingly, they explained that in teeth with shorter roots in the WVC group, the apical and middle thirds are obturated mainly by the single-cone technique since the depth of heat penetration does not often exceed 1‒2 mm. Thus, root length can serve as a confounding factor [ 24 ]. 

On the other hand, in teeth in SC groups (groups 2 and 3), the gutta-percha in the coronal third is vertically packed with a plugger. Therefore, the difference between the samples is expected to be mainly in the middle third of the root. Although the amount of gutta-percha in the apical third of the WVC group (group1) was not different from that in the SC groups (group2 and 3), the sealer further penetrated the canal irregularities in the former technique [ 31 ]. 

Based on recent studies [ 27 - 28 , 32 ], we used micro-CT to evaluate the quality of root filling of different techniques since micro-CT enables quantitative and qualitative analysis of the tooth and other structures [ 33 ]. Furthermore, a study by Hammad *et al*. [ 34 ] indicated the possibility of conducting volumetric measurements of root canal fillings. In the present study, there were no significant differences in the VPs of voids between the groups. However, the VP of the gaps in the apical third was significantly higher than in the middle and coronal thirds in all the canals and obturation groups. This finding can be explained by the fact that the VP of gaps is often higher than voids in all obturation techniques. Similar results were reported in a micro-CT study by Somma *et al*. [ 35 ], who compared the VPs of gaps (internal, external, and hybrid) in single-rooted teeth using the Thermafil, SC obturation, and vertical condensation techniques with AH Plus sealer and reported a higher percentage of gaps (external and combined gaps) compared with voids in all the groups. Another micro-CT study by Celikten *et al*. [ 6 ] also demonstrated a higher percentage of external and combined gaps than internal voids in their study groups. This is probably clinically important because the interfacial gaps in contact with the canal walls have a potential risk of contamination that may eventually lead to leakage. Since interfacial gaps can serve as a gateway for bacterial re-infection of the root canals [ 36 ], emphasis should be placed on finding solutions for root canal system disinfection [ 37 ]. 

Keleş *et al*. [ 20 ] reported a considerable VP of gaps in the apical third. However, they compared the quality of obturation in oval-shaped canals of premolar teeth, used AH Plus sealer with lateral compaction and WVC techniques, and did not provide different definitions for voids and gaps in their study. In contrast, in a micro-CT analysis performed by Celikten *et al*. [ 6 ], a significant reduction in combined gaps was noted in the apical third compared with the middle and coronal thirds in premolar roots obturated by the SC technique. The authors stated that the presence of remarkable anatomical variations in the apical third could explain the lower percentage of combined gaps in the apical third. However, most lateral canals and apical ramifications are found in the apical 3 mm of the roots [ 38 ].

On the other hand, as mentioned earlier, no previous study is available comparing the quality of obturation by the VMC and SC techniques using micro-CT to further compare our results. An *in vitro* design was a limitation of this study. Thus, the results should be generalized to the clinical setting with caution. 

## Conclusion

None of the tested obturation techniques in the present study provided a void-free and gap-free obturation. The apical third of the canals exhibited the highest VP of gaps in all the obturation techniques. 
